# tDCS Over the Motor Cortex Shows Differential Effects on Action and Object Words in Associative Word Learning in Healthy Aging

**DOI:** 10.3389/fnagi.2017.00137

**Published:** 2017-05-15

**Authors:** Meret Branscheidt, Julia Hoppe, Nils Freundlieb, Pienie Zwitserlood, Gianpiero Liuzzi

**Affiliations:** ^1^Department of Neurology, University Hospital ZurichZurich, Switzerland; ^2^Department of Neurology, University Medical Center Hamburg-EppendorfHamburg, Germany; ^3^Brain Stimulation, Department of Psychiatry and Psychotherapy, University Medical Center Hamburg-EppendorfHamburg, Germany; ^4^Department of Psychology, University of MünsterMünster, Germany

**Keywords:** associative word learning, healthy aging, transcranial direct current stimulation, motor cortex, language functions

## Abstract

Healthy aging is accompanied by a continuous decline in cognitive functions. For example, the ability to learn languages decreases with age, while the neurobiological underpinnings for the decline in learning abilities are not known exactly. Transcranial direct current stimulation (tDCS), in combination with appropriate experimental paradigms, is a well-established technique to investigate the mechanisms of learning. Based on previous results in young adults, we tested the suitability of an associative learning paradigm for the acquisition of action- and object-related words in a cohort of older participants. We applied tDCS to the motor cortex (MC) and hypothesized an involvement of the MC in learning action-related words. To test this, a cohort of 18 healthy, older participants (mean age 71) engaged in a computer-assisted associative word-learning paradigm, while tDCS stimulation (anodal, cathodal, sham) was applied to the left MC. Participants’ task performance was quantified in a randomized, cross-over experimental design. Participants successfully learned novel words, correctly translating 39.22% of the words after 1 h of training under sham stimulation. Task performance correlated with scores for declarative verbal learning and logical reasoning. Overall, tDCS did not influence associative word learning, but a specific influence was observed of cathodal tDCS on learning of action-related words during the NMDA-dependent stimulation period. Successful learning of a novel lexicon with associative learning in older participants can only be achieved when the learning procedure is changed in several aspects, relative to young subjects. Learning success showed large inter-individual variance which was dependent on non-linguistic as well as linguistic cognitive functions. Intriguingly, cathodal tDCS influenced the acquisition of action-related words in the NMDA-dependent stimulation period. However, the effect was not specific for the associative learning principle, suggesting more neurobiological fragility of learning in healthy aging compared with young persons.

## Introduction

With increasing life expectancy, quality of life and social participation in older people is more and more dependent on fluid cognitive functioning. However, the ability to acquire new skills, for example to learn a new language, decreases with age (Flöel et al., 2012; Zimerman et al., 2013). The neurobiological aspects underlying language learning in healthy aging constitute a novel research area and are still not well understood. It is known that acquired knowledge and well-trained skills are commonly preserved in healthy aging, while the formation of new memory contents becomes increasingly difficult (Cohen, 1979; Light and Burke, 2009). By consequence, everyday language comprehension shows little abnormalities, whereas linguistic information processing in more challenging situations, for example learning new words, deteriorates with age (Service and Craik, 1993; Light and Burke, 2009).

Associative learning of a new lexicon has been successfully implemented in experimental settings with precise control over stimulus frequency and exposure time (Breitenstein and Knecht, 2002; Dobel et al., 2009; Liuzzi et al., 2010). While these paradigms yielded robust results for different word classes in young adults, testing the suitability and determining learning success in an older population was the scope of the present work.

The framework of embodied semantics is based on the hypothesis that motor areas activated by execution and observation of actions are also involved in processing linguistic information related to these actions (Hauk and Pulvermüller, 2004; Gallese and Lakoff, 2005; Pulvermüller, 2005). Evidence to support this theory comes from fMRI, lesion and electrophysiological studies highlighting a tight functional link between the motor and language systems (Flöel et al., 2003; Kemmerer et al., 2012). Building on this, recent studies have explored the possibility of influencing one system to alter function in the other domain. For instance, it has been shown that listening to food action related sentences results in effector specific excitability changes in the food motor area but not the hand motor area and *vice versa* (Buccino et al., 2005). Also, activation of motor areas (e.g., by allowing manual gestures or suppressing them) can improve certain aspects of language (Rauscher et al., 1996; Pine et al., 2007). Additionally, Liuzzi et al. (2010) could demonstrate that left motor cortex (MC) stimulation was causally involved in learning a novel action word vocabulary. Given the findings for young adults with brain stimulation, we hypothesized an influence of the left MC on the acquisition of a novel action word lexicon. In particular, we explored whether the associative learning principle was specifically altered by brain stimulation in older adults.

Transcranial direct current stimulation (tDCS) is a non-invasive electrical brain stimulation technique, which has been successfully used to improve learning in non-linguistic domains in healthy aging (Hummel et al., 2010; Flöel et al., 2012; Zimerman et al., 2013; Park et al., 2014). While the efficacy of tDCS in improving language function at various levels has been demonstrated in young healthy adults (for an overview see Miniussi et al., 2008; Cotelli et al., 2008), the effect of tDCS on language acquisition in an older population remains an open question. In young people, Liuzzi et al. (2010) demonstrated that tDCS over the left MC affected associative learning of a novel action-word lexicon. tDCS has specific NMDA-dependent and plasticity-related effects that are necessary for the coupling of actions with novel words (Liebetanz et al., 2002; Liuzzi et al., 2010). This allows for a characterization of learning word-to-semantic couplings in a neurobiologically defined way. We here investigated associative word learning of action- and object-related vocabulary applying MC-tDCS in healthy older participants to investigate whether the associative learning principle described in the young is altered in older adults.

We hypothesized that tDCS over the left MC influences associative word learning in healthy, older participants. We were especially interested in whether tDCS has a specific effect on word classes (action- vs. object-related words) and on specific response styles corresponding to Hebbian assumptions.

## Materials and Methods

### Participants

A total of 18 participants were enrolled in the study protocol: 12 females, mean age: 70.6 ± 5.7 years, age range 61–82 years. According to the Edinburgh inventory of handedness, 17 participants were right-handed and one person was born left-handed but retrained to be right-handed during early childhood. All participants were native German speakers and spoke 2.0 ± 1.4 foreign languages. Formal years of education ranged from 8 to 13 years (10.2 ± 1.9).

Participants were not bilingual, had no history of neurological or psychiatric diseases, especially no severe head traumas, seizures, no metal implants in the head/neck region nor pacemaker implantation and did not use neuroactive (e.g., antidepressants, anticonvulsants etc.) or recreational drugs (>6 cups of coffee/day, >50 g of alcohol/day).

To characterize cognitive profiles, participants were screened with a comprehensive battery of neuropsychological tests. Current general cognitive status was assessed with the mini-mental status-test (MMST, Folstein et al., 1975) and the verbal learning and memory test (VLMT, Helmstaedter et al., 2001), verbal fluency with the Regensburg verbal fluency test (Aschenbrenner et al., 2000), visuo-spatial memory and executive abilities with the Rey-Osterrieth complex figure test (Rey, 1959), attention span with the d2-test, working memory with digit spans, and logical reasoning using the Horn Intelligence test (Brickenkamp, 2002). Participants whose performance was more than two standard deviations above or below the age-adjusted mean were to be excluded, but all screened participants were within these boundaries.

This study was carried out in accordance with the recommendations of the local ethics committee at the University of Hamburg and the Deutsche Forschungsgesellschaft (DFG). All subjects gave written informed consent in accordance with the Declaration of Helsinki. The protocol was approved by the ethics committee of the University of Hamburg.

### Stimulus Material

#### Images

An associative word-learning paradigm, previously established and extensively pretested, was used (Breitenstein and Knecht, 2002; Liuzzi et al., 2010; Freundlieb et al., 2012). Participants were presented with spoken pseudowords (e.g., kage, gafo), together with different photographs of actions or objects. For details regarding the generation and compilation of the stimulus material see Liuzzi et al. (2010); Freundlieb et al. (2012).

Actions involving either hands and arms (e.g., knocking or eating), or the whole body (e.g., running or boxing) were taken from a set of photos of everyday actions. Images were previously evaluated regarding quality and suitability for the learning paradigm; assessing naming agreement, quality of depiction, motion association, involvement of a particular body part (head/face/mouth; arm/hand; leg/foot; whole body), daily-life frequency of execution, and frequency of personal everyday performance. Two different pictures were chosen for each action, illustrated by various actors and shot from different perspectives or in different locations (for further details on these images, see Freundlieb et al., 2012).

Object images (e.g., house, tree) were evaluated for recognizability, associations with body parts or motion. Two pictures were selected for each object, depicting the same object concept in two different ways (e.g., different houses). Images were taken from different angles, with different surroundings and without visible human body parts (for details see Freundlieb et al., 2012).

For both visual stimulus sets (action/object images), pictures were converted to grayscale, centered and adjusted for potential distracting features (e.g., text or background objects).

#### Pseudowords

Thirty-four 4-letter pseudowords were taken from an attested language-learning paradigm (Freundlieb et al., 2012). The spoken stimuli (e.g., binu, gafo) complied with the phonotactics of German, had neutral emotional valence and limited associations with existing words. The novel words had a stimulus duration of 970.3 ± 127.4 ms and the same maximum volume. All stimuli were spoken by the same female voice.

### Word Learning Paradigm

During the associative learning task, correct (to be learned) and incorrect picture/pseudoword couplings were presented, with the proportion of correct pairings increasing over time. Participants had to decide intuitively whether word and picture matched or not. Spoken pseudowords were presented over earphones, while pictures were presented on a computer display. The onset of picture presentation was 200 ms after the onset of the spoken pseudoword. Participants answered by left- (correct) or right- (incorrect) clicks on a computer mouse with their right hand. Single-trial duration was 3000 ms, and only responses obtained within this time window were taken into account for analyses. The interstimulus interval (ISI) was 2000 ms. In contrast to the visual presentation duration used in young healthy participants (Freundlieb et al., 2012), we extended their duration (3000 ms) for our cohort of older participants. Pilot experiments showed that most healthy older participants did not learn with short picture presentation times.

**Figure 1:**
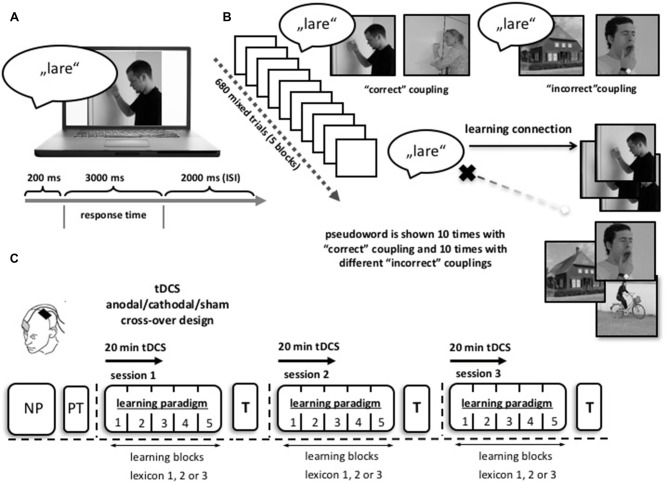
**Word learning paradigm. (A)** Participants were shown a photographic illustration depicting an action/object paired with a spoken pseudoword. They had to decide intuitively if the presented coupling was a correct or incorrect match. Two-hundred milliseconds after onset of the sound, the picture appeared. Responses had to be given within a time window of 3000 ms. The interstimulus interval (ISI) was 2000 ms between two trials. **(B)** One learning session consisted of 680 single trials, subdivided into five blocks with a 2 min break. Each pseudoword was shown 10 times with the “correct” action/object (depicted by two different images) as well as 10 times with different “incorrect” images. **(C)** Study design: prior to the first learning session participants were screened with a neuropsychological test battery (NP) and were introduced to the learning paradigm with a small lexical pre-test (PT) consisting of five words. Each participant completed three learning sessions on three different days with three different lexicon sets. During the task, participants received anodal, cathodal or sham tDCS in a randomized order across learning sessions (1 mA for 20 min; double blind application). At the start of each session the stimulation side over the left motor cortex (MC) was localized using transcranial magnetic stimulation (TMS). tDCS stimulation and the learning paradigm started simultaneously, the latter exceeding the stimulation for approximately 20 min. After each session, patients were asked to translate the acquired pseudowords into notions of their native language (T). Abbreviations: NP, neuropsychological testing; PT, pre-test; T, translation test.

Participants took part in three learning sessions (see description below and Figure 1). During each session, they had to learn 34 pseudowords (17 action- and 17 object-related words), with two different images for each concept. Each session was divided into five blocks, separated by 2 min. breaks. Each block consisted of 136 trials, with a total of 680 trials per learning session. Over the course of the five blocks, correct couplings appeared 10 times (five times for each image of the action/object), whilst each pseudoword was also incorrectly paired with 10 different actions/objects (resulting in a correct/incorrect ratio of 10:1). We ensured that the same auditory/visual pair, as well as the same type of coupling (correct/incorrect) did not occur more than two times in a row.

Every participant completed three sessions on three separate days, with anodal, cathodal or sham tDCS and three parallel lexicon versions (lexicon 1, 2 or 3). The order of interventions and lexicon versions was pseudo-randomized and counterbalanced across sessions. Training sessions were separated by at least 2 and maximum 3 weeks. One week prior to the first tDCS training session, participants were familiarized with the learning paradigm, using a small lexicon of five words. Dependent measures were collected within each session: (1) correct responses during each training session; and (b) translation of pseudowords into German after training. No feedback was given on translation performance (for details see Figure 1).

### Transcranial Direct Current Stimulation

Transcranial magnetic stimulation (TMS) was used to determine the hand region in the left MC in each participant immediately prior to tDCS application. The so-called “hotspot” for the hand region was identified as the position where the highest MEP amplitudes could be consistently evoked in the right first dorsal interosseous muscle (Chen et al., 1998). TMS was delivered by a Magstim 200 stimulator connected to a figure-8 shaped coil (7 cm in diameter, Magstim Co.).

tDCS was administered via two sponge electrodes (Eldith; soaked in 0.9% saline solution) connected to a DC-stimulator (Eldith; serial no. 0006). Either the anode or cathode was placed as stimulating electrode over the left hemispheric “hotspot” of the hand motor area (surface area 25 cm^2^). The reference electrode was positioned over the contralateral supraorbital region (surface area 35 cm^2^). Stimulation started immediately at the beginning of the learning paradigm, and intensity was increased in a ramp-like fashion over 10 s until 1 mA for verum and sham stimulation. In case of anodal or cathodal tDCS stimulation, the current intensity remained constant for 20 min. In contrast, the sham stimulation had a duration period of 30 s during which the intensity was ramped down to zero during the following 10 s. This “fade-in/fade-out” approach is standard best-practise procedure for sham stimulation in tDCS and mimics the cutaneous sensations experienced for verum stimulation (Gandiga et al., 2006). A person not involved in the experiment and data analysis entered the stimulation parameters. Experimenters and participants were blinded for stimulation type.

The neurophysiological effects of tDCS to the MC have been shown to outlast the stimulation period, with effects depending on current intensity and stimulation duration (Nitsche and Paulus, 2000; Nitsche et al., 2003). On this basis, a stimulation period of 20 min was regarded as sufficient for the learning paradigm lasting 40 min.

We evaluated participants’ appraisal of attention, unpleasant sensations (i.e., discomfort/pain) and fatigue with questionnaires using visual analog scales (VAS) as control parameters.

### Data Analysis

Two outcome measures were selected to determine successful learning: (1) the percentage of correct responses in learning blocks over time; and (2) the translation rate for pseudowords into native language after each training session. In a subanalysis, we also calculated the learning success for different response types in each block over time.

To investigate the influence of tDCS, we performed a three-factorial rmANOVA on the dependent variable percentage of correct decisions, with the within-subject factors “stimulation_anodal/cathodal/sham_”, “word class_object/action_” and “blocks_1–5_”. We further investigated how stimulation might change response behavior for the word classes differently by two separate three-factorial rmANOVAs for objects and actions, with the within-subject factors “stimulation_anodal/cathodal/sham_”, “blocks_1–5_” and “response type_hit/miss/corr_reject/false_alarm_”. For translation rates, differences between stimulation sessions were analyzed with a two-factorial rmANOVA, with the within-subject factors “stimulation_anodal/cathodal/sham_” and “word class_object/action_”.

Performance scores of the neuropsychological test battery were probed for linear association with the translation rate and percentage of correct decisions in block five, using Pearson correlation coefficients. A one-way ANOVA with the within-subject factor “stimulation_anodal/cathodal/sham_” was calculated for the VAS outcomes for attention/fatigue/discomfort/pain.

Before application of parametric tests, normal distribution of the dependent variables was tested using Shapiro-Wilk tests and quantile-quantile plots). All ANOVA results were Greenhouse-Geisser corrected if assumptions of sphericity were violated. Paired two-tailed *t*-tests were used for the analysis of the predicted effects of stimulation on word class. Results were considered significant at *p* < 0.05 and Cohen’s *d* is reported as a measure of effect size. All data are expressed as mean ± standard error unless stated otherwise. Statistical analyses were done using SPSS 22.0® and GraphPad Prism® Software.

## Results

### Learning Success

#### Percentage of Correct Responses

**Figure 2:**
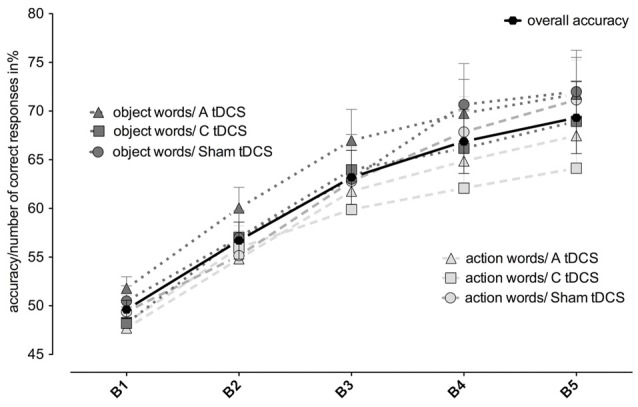
**Changes in accuracy depending on stimulation type and word type**. Mean accuracy scores over the course of five learning blocks (B1–5) split by type of tDCS stimulation (A = anodal = triangles; C = cathodal = rectangles; Sham = circles). Dashed light gray lines depict action words, dotted dark gray lines depict object words; the bold black line with circles depicts overall mean accuracy.

First, we report the effect of tDCS on the performance of associative learning: The number of correct responses increased significantly over the five blocks (blocks_1–5_, *F*_(4,68)_ = 30.44, *p* = 0.000). Averaged over all stimulation conditions, participants started at chance level of 49.33 ± 0.94% and reached 69.32 ± 3.69% in block 5 (see Figure 2). However, anodal and cathodal tDCS stimulation over the left MC did not result in significantly different percentages of correct responses (stimulation_anodal/cathodal/sham_ *F*_(2,34)_ = 1.51, *p* = 0.235; stimulation_anodal/cathodal/sham_ *blocks_1–5_: *F*_(8,136)_ = 1.16, *p* = 0.337), overall mean accuracy in block 5: anodal: 69.61 ± 3.8%; cathodal: 66.54 ± 4.1%; sham: 71.81 ± 4.1%). For all stimulation conditions together, correct responding during learning was better for object- than for action-related words (word class_object/action_, *F*_(1,17)_ = 9.59, *p* = 0.007. There was no significant interaction between blocks and word class (blocks_1–5_* word class_object/action_ *F*_(4,68)_ = 1.09, *p* = 0.368).

Next, we evaluated whether tDCS over the left MC affects word classes differently, as shown in a younger population for cathodal tDCS and action words (Liuzzi et al., 2010). Regarding correct responses, neither the interaction of word class_object/action_ *stimulation_anodal/cathodal/sham_ nor the three-way interaction of blocks_1–5_ *stimulation_anodal/cathodal/sham_ *word class_object/action_ reached significance in the rmANOVA (*F*_(2,34)_ = 2.33, *p* = 0.112, respectively *F*_(8,136)_ = 0.73, *p* = 0.661; see Figure 2). However, as predicted from earlier results in young participants, there was a significant effect of cathodal stimulation on reduction of correct responses of action-related, but not of object-related words (cathodal vs. sham stimulation for actions: 64.1 ± 4.2% vs. 71.2 ± 4.1%, *t*_(17)_ = 2.21 *p* = 0.02, *d* = 0.41, cathodal vs. sham stimulation for objects: 69.0 ± 4.3% vs. 72.9 ± 4.2%, *t*_(17)_ = 1.53, *p* = 0.144, *d* = 0.22).

We also looked at the distribution of correct and incorrect response types (see “Materials and Methods” Section) for the different word classes/stimulation types. There was no interaction of response type_hit/miss/corr_reject/false_alarm_ *stimulation_anodal/cathodal/sham_ nor a three-way interaction of blocks_1–5_ *response type_hit/miss/corr_reject/false_alarm_ *stimulation_anodal/cathodal/sham_ in actions (*F*_(6,102)_ = 479.74, *p* = 0.234, respectively *F*_(24,408)_ = 56.10, *p* = 0.221) or objects (*F*_(6,102)_ = 0.75, *p* = 0.507, respectively *F*_(24,408)_ = 1.12, *p* = 0.358). Note that cathodal stimulation seemed to lead to lower correct rejection and higher false alarm rates for actions compared to objects.

#### Translation

We additionally tested whether participants were able to transfer the learnt association between pseudo-word and images to their native language. Across all stimulation conditions, participants were able to translate a mean of 34.32 ± 5.47% pseudowords into German. tDCS stimulation did not significantly influence overall translation rates (stimulation_anodal/cathodal/sham_: *F*_(2,34)_ = 2.34, *p* = 0.107, overall mean translation: anodal: 32.68 ± 5.1%; cathodal: 31.06 ± 5.5%; sham: 39.22 ± 5.6%). Even though the percentage of correct responses differed for word class, there was no significant difference in overall translation rates (word class_object/action_, *F*_(1,17)_ = 1.15, *p* = 0.299; objects: 51.76 ± 8.1%; actions: 48.24 ± 8.9% of all correctly translated object and action words, respectively). The two-way interaction stimulation_anodal/cathodal/sham_ *word class_object/action_ was also not significant (*F*_(2,34)_ = 2.80, *p* = 0.075, see also Table 1). Our findings suggest that aged-populations might have a limited ability for transfer of associated learning content.

**Table 1:** **Data for the two outcome measures, as a function of stimulation and word class**.

Stimulation	Correct responses (average of last block)		Translation	
	Object	Action	Object	Action
Sham	72.9 ± 4.2%	71.2 ± 4.1%	31.7 ± 6.0%	34.3 ± 6.4%
Anodal	71.7 ± 3.8%	67.5 ± 4.2%	31.7 ± 5.1%	33.7 ± 5.8%
Cathodal	69.0 ± 4.2%	64.1 ± 4.2%	44.1 ± 5.4%	31.4 ± 6.3%

### Neuropsychological Evaluation

**Figure 3:**
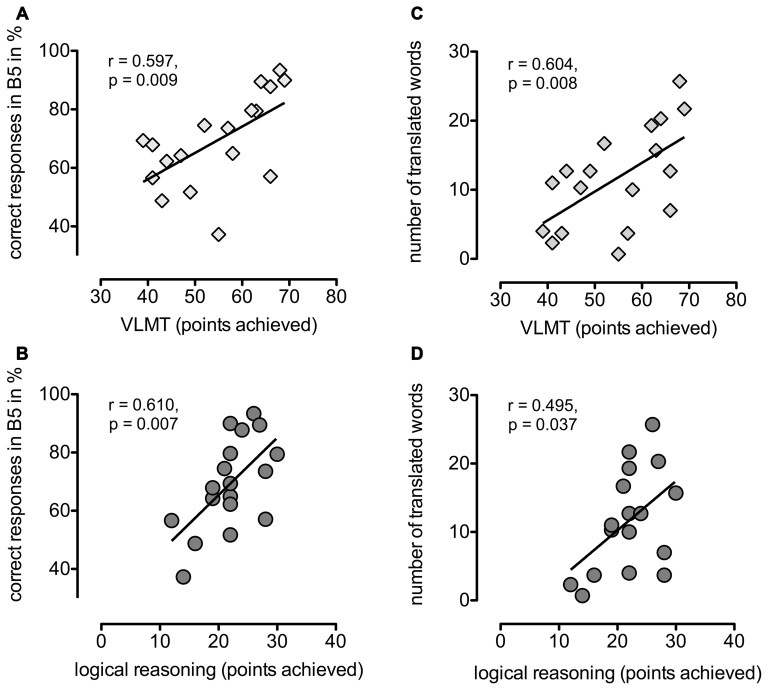
**Cognitive task performance and word learning**. Left side: correlations between learning success measured as mean percentage of correct responses in block 5 and the performance scores of **(A)** VLMT (verbal learning and memory test) respectively **(B)** logical reasoning. Right side: correlations between number of correctly translated words and performance scores of **(C)** VLMT or **(D)** logical reasoning.

Finally, we tried to identify cognitive and stimulation-related factors that could potentially influence a participant’s performance in the associative learning task. Learning success and translation rates correlated with performance scores in the VLMT (verbal learning and memory ability; *r* = 0.597, *p* = 0.009 and *r* = 0.604, *p* = 0.008, respectively) and logical reasoning (*r* = 0.610, *p* = 0.007 and *r* = 0.495, *p* = 0.037, respectively, see Figure 3), while the results for visuo-spatial memory and executive abilities, attention and working memory did not correlate with language learning performance.

Stimulation-related factors did not appear to have a noticeable effect on participant performance. Verum and sham tDCS evoked similar minimal painful sensations (*F*_(2,34)_ = 1.972, *p* = 0.155; mean VAS 1-10 anodal: 1.56 ± 0.2; cathodal: 2.39 ± 0.5; sham: 1.72 ± 0.3). Ratings for discomfort, fatigue and attention were comparable in all groups (discomfort, *F*_(2,34)_ = 0.081; mean VAS 1-10 anodal: 4.06 ± 0.6; cathodal: 3.89 ± 0.5; sham: 3.78 ± 0.5; *p* = 0.923; fatigue, *F*_(2,34)_ = 1.125, *p* = 0.336; mean VAS 1-10 anodal: 5.28 ± 0.7; cathodal: 4.61 ± 0.6; sham: 5.22 ± 0.7; attention, *F*_(2,34)_ = 0.412, *p* = 0.665; mean VAS 1-10 anodal: 3.78 ± 0.5; cathodal: 4.33 ± 0.6; sham: 3.89 ± 0.6).

## Discussion

The aim of this study was to investigate associative word learning and the effect of tDCS over the MC during acquisition of a novel vocabulary in healthy older participants. Using a previously established associative language-learning paradigm (Liuzzi et al., 2010; Freundlieb et al., 2012), older participants acquired a novel vocabulary for everyday objects or actions. No specific effect of anodal or cathodal tDCS application on overall learning success was found, although as predicted there was an impairment of action word acquisition after cathodal tDCS stimulation.

The learning paradigm used here has been applied with some variations in previous studies (Breitenstein and Knecht, 2002; Dobel et al., 2009). Freundlieb et al. (2012) tested the efficiency of the paradigm for learning of novel object and action words in young adults in a single session design, without tDCS stimulation. Paradigm B of their study was similar to our approach except for their shorter visual presentation duration of 1400 ms. Young adults were able to translate 66% of novel words and showed reliable acquisition of the new lexicon, with an overall accuracy rate of 86% in the last block. Performance during the training did not differ between action-related and object-related words, but the translation test revealed significantly better learning of object-related (79%) than of action-related words (53%).

In our study, older participants did not perform as well as the young subjects from Freundlieb et al. (2012) both with respect to translation (66% in young adults, 39% in our older cohort in the sham condition) and correct decisions during training (86% correct decisions in the last block in young adults, 71% in our older population in the sham condition). Additionally, pretesting showed that learning in the older participants was only possible when picture presentation time was more than doubled compared with the duration for young participants. We cannot draw any firm conclusions about which specific process is most compromised in the older participants. Aging may impair both non-linguistic (visuospatial and auditory processing, working memory) as well as linguistic functioning (phonological processes, binding phonological information with semantic context). It can only be concluded that associative word learning is relatively preserved, but is considerably slowed down by aging.

Liuzzi et al. (2010) evaluated the functional role of the MC in action-word acquisition in young healthy participants. Using the same associative learning task that coupled spoken words with action-related pictures only, participants learned one set of 76 pseudowords during four consecutive training sessions. Either anodal, cathodal or sham tDCS stimulation over the MC was administered for 20 min prior to each session. Differences between their and our associative-learning paradigm concerned the number of novel words (76 overall vs. 34 per session), the use of action pictures or action and object pictures, visual presentation time (1400 vs. 3000 ms), the ratio between correct and incorrect couplings (4:2 vs. 10:1), a longer training duration (with training sessions on four consecutive days, with learning success measured on day four vs. three different sets and learning assessments each day) and the design (anodal, cathodal, sham between vs. within subjects). Liuzzi et al. (2010) could demonstrate that cathodal tDCS to the left MC led to a significantly reduced translation of novel action words compared to sham stimulation, whereas no effect was seen for anodal stimulation. Control experiments with object-related pseudowords or stimulation over prefrontal areas indicated a semantic and topographic specificity of the observed effect after cathodal tDCS over the MC (Liuzzi et al., 2010).

In older participants, we also observed that action-word acquisition after cathodal tDCS was significantly lower compared to the sham group. However, the effect was weaker compared with our previous study in young participants. This might be due to the single-session crossover design vs. repeated training over 4 days. Furthermore, while the study design of Liuzzi et al. (2010) allowed for consolidation effects overnight, the older participants were tested on the same day. It has been shown that the time for memory consolidation can have a crucial influence on novel word acquisition in an associative learning paradigm (Geukes et al., 2015). The effect of cathodal tDCS could only be shown in the NMDA-dependent stimulation period, supporting the idea that the MC is functionally connected during learning of novel action words. The pattern of results makes other effects like shifts of membrane polarization during the initial 20 min of stimulation less likely, given that inadvertent general effects on word learning could also not be shown in this study. As in young participants, we could not enhance learning with anodal tDCS over the MC.

In our participant group, verbal-learning ability showed good correlation with successful lexical acquisition. This finding is in line with previous findings in young healthy participants that showed a positive correlation of verbal-memory abilities with associative object word learning (Breitenstein and Knecht, 2002). More intriguing was the finding that logical reasoning and associative word learning were correlated. This is consistent with findings in aphasic patients, suggesting that preservation of executive functions may promote therapeutic outcome (Fillingham et al., 2005, 2006; Nicholas et al., 2005; Fridriksson et al., 2006). Different fMRI studies have shown that besides some task-specific regions decreasing in activity, especially prefrontal activation increases in older adults (Park and Reuter-Lorenz, 2009). The correlation of executive cognitive functions such as logical reasoning with lexical acquisition could hence suggest a compensatory strategy to maintain behavioral performance.

To summarize, associative learning imposes minimal demands on conscious effort compared with declarative vocabulary learning. This makes computer-based associative learning paradigms a promising tool for language learning in healthy aging. The ability for semantic transfer seems to be compromised in older people. Thus, training needs to be more intense in frequency and presentation times in order to build stable word representations.

Non-invasive brain stimulation techniques such as tDCS can be used to probe the interaction of specific brain areas with cognitive performance, to gain a better understanding of age-related changes of learning. Beyond this, the thorough knowledge of age-dependent cognitive skills in healthy older people might help to find predictive factors for language recovery in aphasic patients and to improve speech and language therapy.

## Author Contributions

GL: study conception and design. MB, JH, GL: acquisition, analysis and interpretation of data. MB, JH, GL: drafting the manuscript. GL, PZ, NF: critical revision. MB, JH, NF, PZ, GL: final approval of the version to be published and agreement to be accountable for all aspects of the work.

## Funding

The present study was supported by the Deutsche Forschungsgemeinschaft (DFG LI 1892/1-1 to GL).

## Conflict of Interest Statement

The authors declare that the research was conducted in the absence of any commercial or financial relationships that could be construed as a potential conflict of interest.

## Abbreviations

ANOVA: analysis of variance
ISI: interstimulus interval
MC: motor cortex
MEP: motor evoked potential
MMST: mini-mental status-test
tDCS: transcranial direct current stimulation
TMS: transcranial magnetic stimulation
VAS: visual analog scales
VLMT: verbal learning and memory test.
